# Highly Transparent and Flexible Iontronic Pressure Sensors Based on an Opaque to Transparent Transition

**DOI:** 10.1002/advs.202000348

**Published:** 2020-03-12

**Authors:** Qingxian Liu, Zhiguang Liu, Chenggao Li, Kewei Xie, Pang Zhu, Biqi Shao, Jianming Zhang, Junlong Yang, Jin Zhang, Quan Wang, Chuan Fei Guo

**Affiliations:** ^1^ Department of Materials Science and Engineering Southern University of Science and Technology Shenzhen 518055 China; ^2^ Department of Mechanics and Aerospace Engineering Southern University of Science and Technology Shenzhen 518055 China; ^3^ Department of Computer Science and Engineering Southern University of Science and Technology Shenzhen 518055 China; ^4^ Department of Civil and Environmental Engineering Shantou University Shantou Guangdong 515063 China

**Keywords:** electric double layers, flexible pressure sensors, refractive index, smart windows, transparent bands, transparent pressure sensors

## Abstract

Human–computer interfaces, smart glasses, touch screens, and some electronic skins require highly transparent and flexible pressure‐sensing elements. Flexible pressure sensors often apply a microstructured or porous active material to improve their sensitivity and response speed. However, the microstructures or small pores will result in high haze and low transparency of the device, and thus it is challenging to balance the sensitivity and transparency simultaneously in flexible pressure sensors or electronic skins. Here, for a capacitive‐type sensor that consists of a porous polyvinylidene fluoride (PVDF) film sandwiched between two transparent electrodes, the challenge is addressed by filling the pores with ionic liquid that has the same refractive index with PVDF, and the transmittance of the film dramatically boosts from 0 to 94.8% in the visible range. Apart from optical matching, the ionic liquid also significantly improves the signal intensity as well as the sensitivity due to the formation of an electric double layer at the dielectric‐electrode interfaces, and improves the toughness and stretchability of the active material benefiting from a plasticization effect. Such transparent and flexible sensors will be useful in smart windows, invisible bands, and so forth.

## Graphical abstract

An opaque to transparent transition happens when a microporous dielectric is filled with ionic liquid with a close refractive index. For a capacitive‐type pressure sensor with a porous dielectric, the filling of ionic liquid can significantly improve its transparency to 90%, and enhance its sensitivity by introducing electric double layers, thus enabling wide applications.

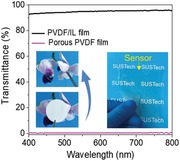

Flexible pressure sensors and electronic skins have been gaining increasing attentions due to their promising applications in human–machine interaction,^[^ ^1^, ^2^ ^]^ soft robotics,^[^ ^3^ ^]^ human health monitoring,^[^ ^4^ ^]^ etc. The fast development of artificial intelligence brings a promising but challenging opportunity for these sensors: combined properties of high transparency and high sensitivity are required for applications in smart windows, invisible robots, and touch screens. Capacitive‐type flexible pressure sensors are an ideal selection toward real applications because they present advantages of low signal drift and simple device structure,^[^ ^5^, ^6^, ^7^, ^8^ ^]^ but they also exhibit limited sensitivity due to the limited compressibility of the soft dielectric layer. Micropores or surface microstructures are often introduced to the dielectric to improve the compressibility and thus enhance the sensitivity of the device.^[^ ^9^, ^10^, ^11^ ^]^ The microstructures, however, strongly scatter light such that the dielectric layer presents a high haze and low transparency.^[^ ^12^ ^]^ It is thus a big challenge to balance high sensitivity and high transparency in a capacitive‐type flexible pressure sensor.^[^ ^13^, ^14^ ^]^

The light scattering caused by the microsurfaces or porous structures stems from the large difference in refractive index (*n*) of the rough interface between the matrix material and air.^[^ ^15^ ^]^ Selecting a filler whose refractive index matches well with that of the matrix to replace the air in the microstructured or porous materials would be an effective way to keep interface continuity and thus reduce light scattering.^[^ ^16^ ^]^ Here we used a microporous polyvinylidene fluoride (PVDF) membrane, a cost effective and commercially available material as the dielectric and employed an ionic liquid (IL) that has the same *n* (*n* = 1.41) with that of PVDF. Filling the pores with the IL results in a dramatical increase in optical transmittance (*T*) from 0 for the initial porous PVDF film to 94.8% for the PVDF/IL membrane by matching the refractive index and by smoothening the microstructured surface. The IL also plays two other important roles. First, it offers migratory ions to form an electric double layer (EDL) at the dielectric/electrode interface, for which the positive and negative charges are separated at a distance of ≈1 nm to have a huge capacitance density of *C*_EDL_ ≈ 10^−1^ F m^−2^.^[^ ^17^, ^18^ ^]^ Second, the IL significantly enhances the toughness of the porous PVDF membrane due to a plasticization effect that improves its flexibility and mechanical stability. A pressure sensor applying the IL‐filled porous PVDF membrane exhibits high flexibility, a transparency of 90.4%, a sensitivity of 1.19 kPa^−1^, and a low detection limit of ≈0.4 Pa, well combining the flexibility, transparency, and sensing properties. The approach by filling *n*‐matching liquid to microporous or microstructured dielectric to improve its optical transparency and sensing performance is simple and general, and is expected to be extended to other material systems, as well as other applications such as transparent batteries, supercapacitors, etc.

We fabricated the flexible and highly transparent dielectric material by simply immersing a piece of porous PVDF membrane into IL (1‐butyl‐3‐methylimidazolium hexafluorophosphate, [BMIM]PF_6_), as illustrated in **Figure** **1**a. The porous PVDF film, which is fully opaque before loading the IL, becomes highly transparent after filling with the [BMIM]PF_6_ IL (insets of Figure 1b). Our experimental data reveal that the transmittance of the PVDF/IL composite membrane significantly changes from 0 to 94.8% at 550 nm (Figure 1b), even higher than the transmittance of common glass. Because of the large capillary force at microscale,^[^ ^19^ ^]^ IL will spontaneously infiltrate into the pores once the porous PVDF film is immersed in IL (Figure S1, Supporting Information). Such an opaque to transparent transition finishes within 1 s (as illustrated in Movie S1, Supporting Information). This simple and efficient process can be used to make large‐scale transparent PVDF/IL composite membrane, exemplified by the one with a scale of 30 cm × 16 cm (Figure 1c).

**Figure 1:**
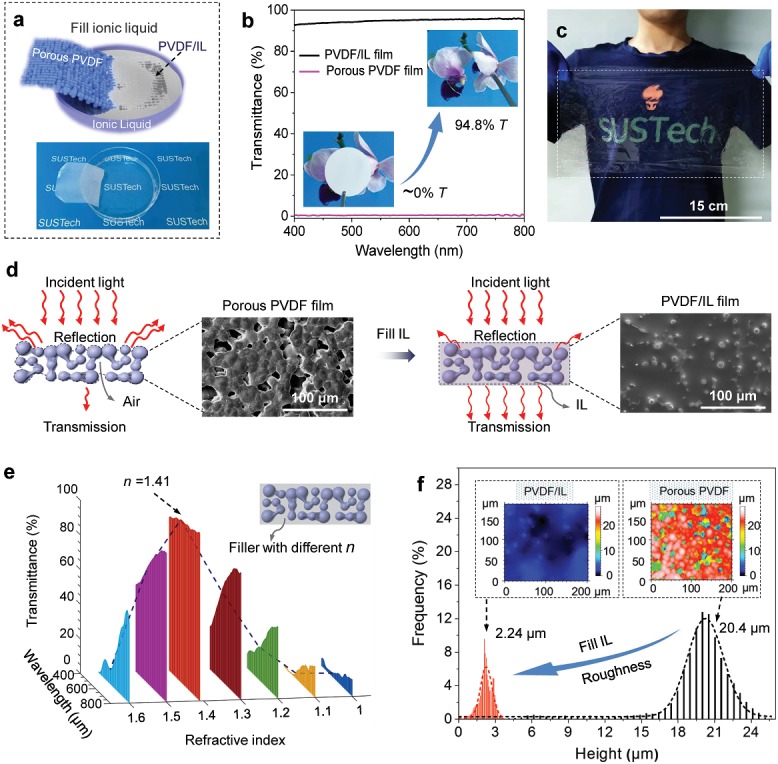
Preparation, transmittance, and opaque to transparent transition mechanism of the flexible and transparent PVDF/IL composite film. a) Schematic diagram (top) of the fabrication of flexible and transparent PVDF/IL composite film, and a photo (bottom) showing that the PVDF film turns highly transparent immediately after being immersed in IL. b) Transmittance spectra of the original porous PVDF film and the PVDF/IL composite film in the wavelength range of 400–800 nm; insets are corresponding digital photos. c) A PVDF/IL composite film with a dimension of 30 cm × 16 cm. d) Schematic diagram for the opaque to transparent transition mechanism. Insets are SEM images of the porous PVDF film before and after filling IL. e) Optical simulation of porous PVDF films that are filled with fillers with various refractive indices. Here *n* represents refractive index. f) Height distribution of the porous PVDF film and the PVDF/IL film. Insets are three‐dimensional optical images showing significantly different roughness.

Ionic liquids are difficult to volatilize due to their negligible vapor pressure and higher density compared with those of aqueous and organic solvents.^[^ ^20^, ^21^ ^]^ Therefore, ionic liquid can exist stably in the porous PVDF film and has no obvious volatilization under relatively mild conditions. It is worth pointing out that the porous skeleton can also stably keep the IL under compression because of the high elastic modulus of porous PVDF (≈280 MPa, which is 3–4 orders magnitude higher than the applied stresses) and the great capillary tension at microscale. Figure S2 (Supporting Information) shows that the mass loss of the IL is only ≈3.6% after 100 compression/release cycles under a high pressure of 120 kPa when the PVDF/IL composite film is placed on an oleophobic substrate. The mass loss is caused by that the IL on PVDF surface will be adherent on the substrate when a pressure is applied. This problem can be solved by sandwiching the PVDF/IL composite film with two pieces of clean substrates and applying a preload to remove part of the IL on PVDF surface.

The mechanism for the opaque to transparent transition is illustrated in the schematic diagram of Figure 1d. The initial porous PVDF film consists of dense micropores and crystallized microparticles of 10–20 µm in diameter. This morphology is caused by PVDF crystallization and solvent volatilization in a phase separation process.^[^ ^22^, ^23^ ^]^ After loading IL, the pores of porous PVDF film are filled with IL, leaving a relatively smooth surface. The opaque to transparent transition of the porous film lies in two roles of the IL filler. First and most importantly, the IL has a refractive index (*n*_IL_ = 1.41) quite close to that of PVDF (*n*_PVDF_ = 1.42). The match of refractive index means that the PVDF/IL composite membrane is a homogeneous medium for light, thereby light scattering at the interface does not occur and the composite will be highly transparent. A finite elemental method is implemented to confirm the effect of *n*‐matching and the results is shown in Figure 1e. At the initial state, the pores of the porous PVDF film are filled with air, forming an aerogel. The *n* of the pores is set to be 1 to simulate air under the standard condition which is far smaller than that of PVDF. The porous PVDF film is therefore an inhomogeneous medium that suffers from large interface light scattering, causing a significant loss of optical transparency. The transmittance of the PVDF/filler composite film is improved as the refractive index of the filler gradually gets closer to that of the PVDF substrate. When *n* of the filler (*n*_filler_) is set to 1.41 that equals to the refractive index of [BMIM]PF_6_, or *n*_filler_ *= n*_PVDF_, the transmittance boots to 93.7% at 550 nm, which is close to our experimental result. As *n* of the filler further increases to larger than *n*_PVDF_, the transmittance decreases due to increased interface scattering caused by *n* mismatch. Experimental results shown in Figure S3 (Supporting Information) further proves the effect of *n*‐matching on optical transparency of the porous PVDF film by filling various colorless liquids including water (*n* = 1.333), simethicone (*n* = 1.406), BMIM]PF_6_ (*n* = 1.410), and glycerinum (*n* = 1.473). It is worth pointing out that high interconnection is necessary for the IL to fully fill the pores. Closed pores in the PVDF substrate will not be filled with liquid but act as light scattering centers (schematic as shown in Figure S4, Supporting Information).

Second, the IL significantly smoothens the microstructured surfaces of the porous PVDF film and thus reduces the light scattering at the air/composite film interface. The microstructures in the porous PVDF film generate large roughness which induces severe light scattering according to the Lorentz–Mie theory.^[^ ^24^ ^]^ After loading IL, the pores are filled up to maintain a relatively smooth surface by the capillary tension. The height distribution and quantitative statistical information of the initial porous PVDF film and the PVDF/IL composite film are presented in Figure 1f. It shows that the porous PVDF film has a broad height distribution in the range from 16.5 to 24 µm and is centered at 20.4 µm, indicating a large surface roughness. By contrast, the height difference of the PVDF/IL composite film is greatly decreased to 2.24 µm with a much narrower distribution between 0 and 3.8 µm. In addition, the number of surface microstructures of the PVDF/IL film also decreases because the microstructures with relatively lower altitudes are immersed by the IL. Such changes will help reduce the light scattering greatly. The light scattering coefficient (*b*_sp_) of a rough surface can be expressed as^[^ ^25^ ^]^

(1) $$b_{\text{sp}}=\int \frac{\pi D_{\text{p}}^{2}}{4}\;Q_{\text{s}}\left(m,D_{\text{p}},\lambda \right)n\left(D_{\text{p}}\right)\text{d}D_{\text{p}}$$

where *D*_p_ is the diameter of the microstructure, *Q*_S_ is the single microstructure scattering efficiency, and *n*(*D*_p_) is the microstructure number distribution, and this equation indicates that light scattering decreases with the decreasing of structure size and number, well explaining our observed results. There are still a few protrusions in the surface but cause little adverse effect to the transmittance. Such protrusions, on the other hand, are quite useful to achieve a high sensitivity of sensors, which will be discussed later.

Such an opaque to transparent transition mechanism can also be extended to other porous material systems. For instance, porous cellulose acetate (CA) (*n* ≈ 1.48) film and porous polylactic acid (PLA) (*n ≈* 1.45) film could also become transparent when immersed in ILs with different refractive indices, including [BMIM]PF_6_, 1‐butyl‐3‐methylimidazolium chloride ([BMIM]Cl), and 1‐ethyl‐3‐methylimidazolium bis(trifluoromethylsulfonyl)imide ([EMIM]TF_2_N) for which *n* is 1.41, 1.49, and 1.43, respectively, as indicated in **Figure** **2**a,b. Similarly, smoothed surfaces are also seen in these systems (Figure 2c,d). This opaque to transparent transition also works in other systems without using IL. For example, we can make polytetrafluoroethylene (PTFE, *n* ≈ 1.35) highly transparent by filling normal saline (*n* ≈ 1.33), as shown in Figure S5 (Supporting Information). Our solid experimental and simulated results verify that utilizing *n*‐matching ionic conductors to fill porous materials is an effective approach to improve their transparency while maintaining the mechanical structures. The transparent ionic films can then be used as the dielectric for highly transparent iontronic pressure sensors.

**Figure 2:**
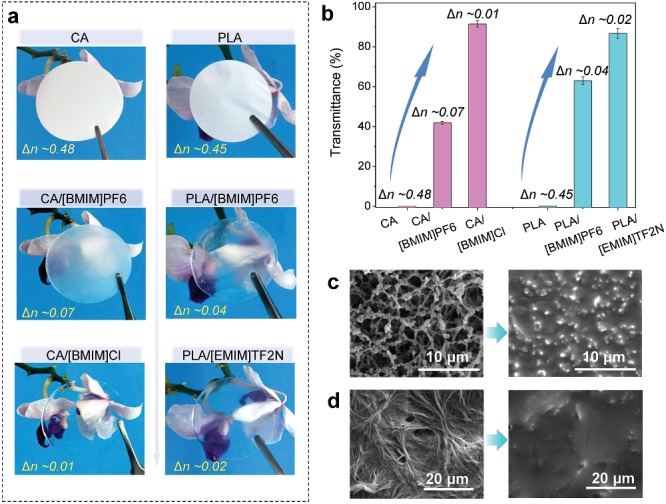
Preparation of various transparent dielectric by filling *n*‐matching IL in microporous materials. a) Digital photos of porous materials of cellulose acetate (CA) and polylactide (PLA) before and after filled with various IL, the difference in refractive index (Δ*n*) is indicated. b) The transparency increases as *n*_IL_ gets close to that of the CA or the PLA substrate. SEM images of the microporous c) CA film and d) PLA film before and after filling with IL.

The capacitive‐type pressure sensor in this work consists of two flexible transparent electrodes and the PVDF/IL film between them (**Figure** **3**a). The flexible transparent electrodes are critical to achieve high transparency of the device. Ultrathin silver nanowire (AgNW) films, which exhibit high transparency as well as high flexibility,^[^ ^26^, ^27^, ^28^ ^]^ were used as the flexible transparent electrodes in the sensors. AgNWs having an average diameter of ≈30 nm and an average length of ≈30 µm were spray‐coated on the surface of a thin polyimide (PI) film (Figure S6, Supporting Information). The transmittance of the AgNW network film is 97.6% at a sheet resistance of 80 Ω sq^−1^. Although the transmittance of the pressure sensor is lower than that of the PVDF/IL film because the AgNWs electrodes will cause extra transparency loss, it still reaches a high value of 90.4% (Figure 3a). From a digital photo of Figure 3b we can see that our pressure sensor is high transparent and can be bent easily since both the PVDF/IL layer and the AgNWs electrodes are highly flexible and transparent.

**Figure 3:**
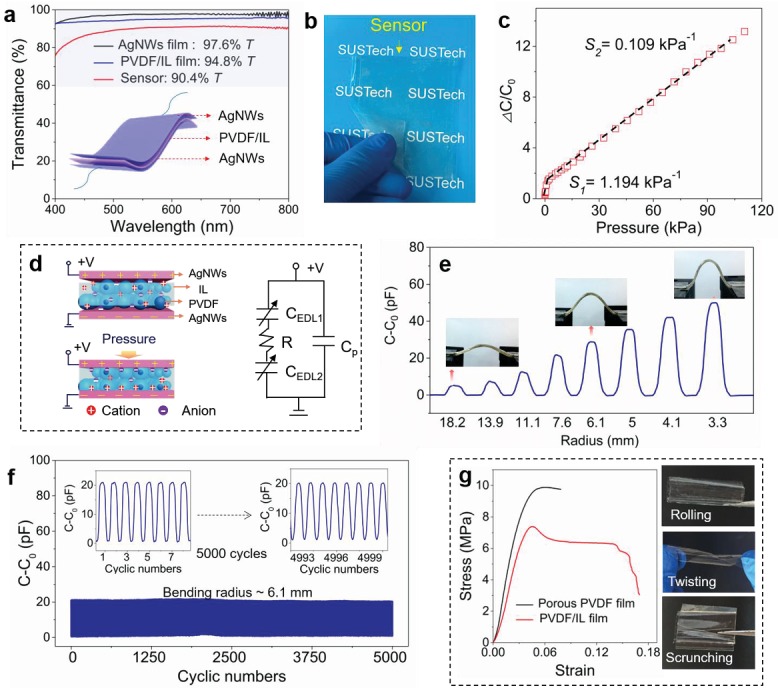
Transmittance, sensitivity, and mechanical stability of the flexible transparent pressure sensor. a) Transmittance spectra of AgNWs electrode (80 Ω sq^−1^), PVDF/IL dielectric layer, and the pressure sensor. Inset illustrates the structure of the capacitive pressure sensor. b) Optical image of the flexible transparent sensor. c) Normalized change in capacitance as a function of applied pressure of the sensor. d) Schematic showing the sensing mechanism of the sensor and its equivalent circuit. e) Capacitance responses of the flexible transparent pressure sensor under various bending radii. f) Signal of the sensor subjected to 5000 bending/release cycles under a bending radius of ≈6.1 mm, showing high stability over cycles. g) Stress–strain curves of the initial porous PVDF membrane and the PVDF/IL composite membrane. Insets show the PVDF/IL composite film under rolling, twisting, and folding.

The sensitivity (*S*) of a capacitive pressure sensor can be expressed as *S* = δ(Δ*C*/*C*_0_)/*δP*, where *C*_0_ is the initial capacitance, Δ*C* is the relative change of capacitance, and *P* is the applied pressure.^[^ ^29^ ^]^ The sensitivity is figured out according to the formula by loading a pressure *P* on the sensor and recording the relative change in capacitance. Figure 3c exhibits that the sensitivity of our sensor is 1.194 kPa^−1^ under a low‐pressure range (0–0.5 kPa) and a linear sensitivity of 0.109 kPa^−1^ over a broad pressure range (0.5–120 kPa). The measured sensitivities are significantly higher than that of a control sensor using porous PVDF as the dielectric layer (with a maximum sensitivity of only 0.245 kPa^−1^, Figure S7, Supporting Information). The response to applied pressure comes from the change in contact area of the IL/electrode interface, at which an EDL forms, as illustrated in Figure 3d. Because of the formation of EDL, the capacitance density also enhances from ≈10 pF cm^−2^ to ≈0.1 nF cm^−2^, and the enhanced capacitance density is helpful to achieve a higher signal to noise ratio. We need to point out all the materials we used in the sensor are commercially available. The porous PVDF membrane might be replaced by other materials to achieve a higher sensing performance. However, the porous PVDF is still an idea selection because it is cost‐effective and easy to fabricate.

The PVDF/IL composite film plays several important roles. First, it serves as a viscoelastic framework that responds almost elastically to the external force because of the high elastic modulus of PVDF, and the low‐viscosity IL will cause only a slight delay to the response. Second, the IL trapped in the pores of the PVDF film offers free ions to form EDL, for which the negative and positive charges are in nanometer separation and thus the capacitance density is superlarge (5–6 orders larger than the capacitance density of a direct capacitor). The total capacitance of the device can be calculated according to the equivalent circuit displayed in Figure 3d: the top and bottom EDL capacitors (*C*_EDL1_ and *C*_EDL2_) are connected in series, and then in parallel with a direct capacitor which can often be ignored since it is much smaller than the EDL capacitance. Before loading, the electrodes are supported by only a few residual surface protuberances of the PVDF/IL film, such that the initial capacitance is small. As the load increases, the contact area of the interface between the IL and the electrodes increases as a function of the load, resulting in an enhanced EDL capacitance of the interface. Besides, our flexible transparent sensor also exhibits a fast response time of 40 ms (comparable to that of the human skin) and a low limit of detection as low as 0.4 Pa (Figure S8, Supporting Information). The fast response speed lies in the microstructured PVDF but is partially slowed down by the viscous IL filled in the pores. The viscoelastic nature of the film can be verified in Figure S9 (Supporting Information), which shows that our sensor has a small hysteresis. The sensor also has a remarkable frequency‐dependent and temperature‐dependent response (Figure S10, Supporting Information), which is a typical phenomenon in iontronic devices.^[^ ^30^ ^]^

The sensor also presents high mechanical durability over loading/release cycles. For example, no change in the signal amplitude was found when subjected to 5000 compression/release cycles under a peak pressure of ≈10 kPa (Figure S11, Supporting Information). In addition, bending test was also implemented to evaluate the flexibility of our sensor. As shown in Figure 3e, the capacitance of our sensor increases as the bending radius decreases. This is because decreasing bending radius will generate larger compressive strains at the EDL interface. The signal returns to the initial value upon releasing, and exhibits negligible variation over 5000 bending/release cycles under a bending radius of ≈6.1 mm (Figure 3f). All these experimental data verify that our sensor is highly flexible and has little fatigue under cyclic loading/unloading. Note that bending will cause a “piezoionic effect.”^[^ ^31^ ^]^ Here in this work, the measurement voltage is constant (1 V), which is much larger than the voltage (≈1 mV) generated by the piezoionic effect. Therefore, the interference caused by the piezoionic effect can be ignored.

The high mechanical stability can be ascribed to the improved toughness of PVDF with the addition of the IL. We can see from Figure 3g that the PVDF/IL composite film can be stretched up to a strain of 0.141, which is twice that of the initial porous PVDF film without IL. The toughness of the PVDF/IL composite film can be deduced from the area under the stress–strain curve and is determined to be 960.3 kJ m^−3^, which is 66.7% higher than that of the initial porous PVDF film (576.3 kJ m^−3^). On the other hand, both the elastic modulus and the yield strength of the PVDF/IL composite film are lower than those of the initial porous PVDF film due to an IL‐induced plasticization effect that causes significant energy dissipation.^[^ ^32^ ^]^ The plasticization effect can be confirmed by rupture analysis shown in Figure S12 (Supporting Information), for which the fracture surface of the PVDF/IL composite film has oriented strips along the tensile direction caused by a significant shear yielding.^[^ ^33^ ^]^ Such a softer material with improved ductility and larger toughness is desired for flexible electronics, and we have verified in Figure 3g that the film can be significantly rolled, twisted, or scrunched without rupture.

The features of high transparency and high sensing performances allow our flexible pressure sensor to be applied in human machine interaction, wearables, flexible displays, and smart windows. Here we demonstrate its application as a transparent wearable band and a smart window. As illustrated in **Figure** **4**a, a flexible transparent 3 × 4 sensor array was laminated on a wrist as a band, and the pixels (each sensor is a pixel) were designed to mimic phone keys, including ten numbers from 0 to 9, a star and a pound key (bottom right of Figure 4a). Each pixel presents a large change in signal amplitude upon finger touch, and the dialed numbers are recorded by using a data acquisition circuit (upper right of Figure 4a). The capacitance signals are then switched to voltage signals and transferred to a mobile phone by Bluetooth. The signal switching paths are shown in Figure 4b. We have successfully used our wearable flexible transparent keyboard to make a dial call, as shown in Figure 4c and Movie S2 (Supporting Information). Such a flexible and transparent wearable keyboard is promising in human–machine interaction and mobile devices.

**Figure 4:**
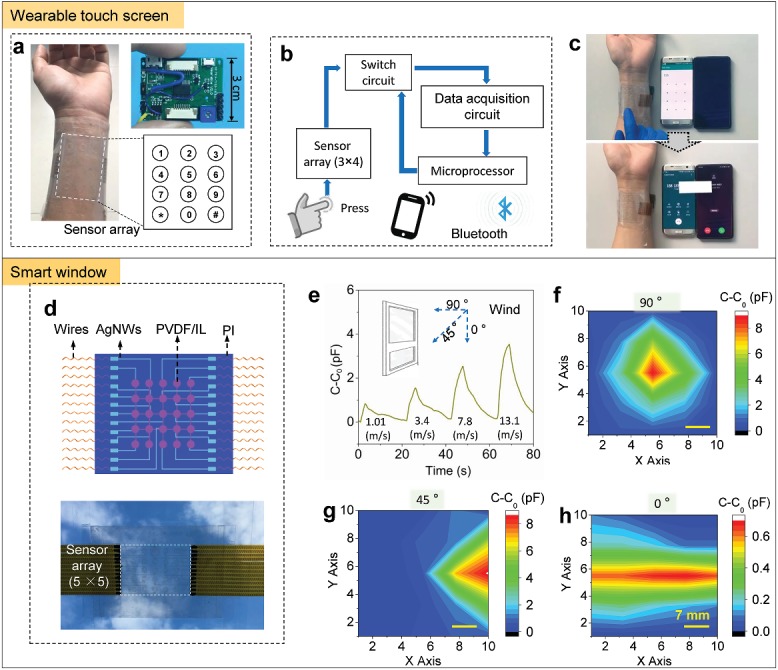
Applications of the transparent and flexible pressure sensor array in a smart band and a smart window. a) Photograph of the wearable smart band with 3 × 4 pixels. Upper right schematic shows dial numbers corresponding to the pixels, and the bottom right photo is a printed circuit board that connects to the pixels for signal collection and transmission. b) Schematic diagram of signal transmission paths from the sensor array to mobile device. c) Dialing with the smart band. Phone numbers are blacked out. d) Schematic illustration (top) and a photo (bottom) of a 5 × 5 pixel array for smart window. e) Detection of wind speeds with the smart window. f–h) Mapped signals of the sensor array under various wind direction of 90°, 45°, and 0°, respectively. Scale bars are 7 mm.

The highly transparent sensor array sensor can also be used for smart glass that offers the capability to detect the intensity and direction of wind. We have fabricated a flexible and transparent sensor array with 5 × 5 pixels and attached the array on a piece of glass to evaluate its feasibility as a smart window. The schematic diagram of fabrication process of sensor array is shown in Figure S13 (Supporting Information) and the structure of the sensor array is shown in Figure 4d. The test is carried out by blowing to the smart window with various wind speeds and recording the capacitance signals of one sensing element. As demonstrated in Figure 4e, our sensor shows significantly different signal amplitudes at wind velocities of 1.01, 3.4, 7.8, and 13.1 m s^−1^, with the peak amplitude increasing along with the increasing wind velocity. In addition, wind direction could be identified by using the smart window. The mapping of the capacitance amplitude of all pixels is able to precisely reflect wind directions that are 90°, 45°, and 0° to the window plane (Figure 4f–h). Those results indicate that the pressure sensor array has a potential to be used in the smart windows due to its capability to sense wind velocity and direction.

In summary, this work introduces a general strategy to make transparent flexible pressure sensors with a microporous dielectric by filling an *n*‐matching ionic liquid in the pores of the microporous dielectric. The ionic liquid plays a few important roles. First, the match of the refractive index between the porous materials and the ionic liquid homogenizes the optical medium and removes light scattering, achieving a transmittance up to 94.8%. Second, the ions offer EDL capacitance that significantly improves the signal intensity and sensitivity of the sensors. Third, the ionic liquid improves the flexibility (including improved toughness and ductility, as well as reduced rigidity) of the dielectric by means of plasticization, and thus the sensors have high mechanical stability over loading/unloading cycles. We have demonstrated that the transparent flexible sensor is potentially useful as on‐skin touch screens and smart windows, and expected to be applied in human–machine interfaces, electronic game industry, as well as pressure sensing under water.

## Experimental Section

##### Fabrication of Porous Materials

PVDF with an average number molecular weight of about 100 kg mol^−1^ was purchased from Yuyao Suhe Plastics Co. Ltd, China. First, a certain amount of PVDF was dissolved in *N*‐*N*‐dimethylformamide (DMF) at a temperature of 80 °C to form a solution with a PVDF concentration of 100 mg mL^−1^. Next, the solution was casted on a clean glass plate to form a liquid film with a thickness of ≈0.2 mm, and the liquid film was immediately transferred to an oven with a preset temperature of 50 °C for 4 h, forming a porous PVDF membrane with the evaporation of DMF and PVDF crystallization. Levorotatory PLA was provided by Changchun Sinobiomaterials Co., Ltd, China, having a viscosity‐average molecular weight of 193 kg mol^−1^. PLA was dissolved in DMF at 80 °C to form a casting solution, and PLA concentration in the solution was 100 mg mL^−1^. The solution was then casted between two clean glass plates to form a sealed sandwich structure. Immediately, this specimen was placed in a refrigerator at a temperature of −18 °C for 2 h to form a PLA gel film. Finally, a porous PLA film was obtained by immersing the PLA gel film in water for overnight followed by subsequent evaporation of the water at room temperature. CA films with a pore size of about 0.8 µm were purchased from Shanghai Xin Ya Purification Equipment Co., Ltd, China and used as received. PTFE fiber films were supplied by Suzhou Youkefa New Material Technology Co., Ltd, China. The pore size of the PTFE was about 1 µm and the thickness was 20 µm. Because PTFE was hydrophobic, the PTFE film was presoaked in ethanol and then immersed in normal saline for full infiltration.

##### Fabrication of the Flexible and Transparent Dielectric

The ILs used in this work including [BMIM]PF6, [BMIM]Cl, and [EMIM]TF2N were all purchased from Lanzhou Institute of Chemical Physics, China. The flexible and transparent dielectric materials were made by simply immersing the porous membranes in IL or solution at ambient temperature. The PVDF/IL film would be covered with a piece of dustless cloth to remove part of the surface IL before it was used in a sensor. IL or other colorless liquid infiltrated into the porous film due to the capillary force provided by the pores. Finally, redundant IL was removed through standing or wiping the surface of the composite film.

##### Fabrication of Flexible and Transparent Electrodes and Sensors

Transparent electrodes were fabricated by spray coating AgNWs on the surface of transparent PI film. AgNW suspension with an average nanowire diameter of ≈30 nm and an average length of ≈30 µm was purchased from Xianfeng Nanomaterials Technology Co., Ltd. The AgNW suspension was modified by adding hydroxypropylmethyl cellulose (HPMC 0.5 wt%) serving as an adhesive. After spray coating, the films were dried at 80 °C for 20 min. The AgNWs exhibit strong adhesion to the PI substrate due to the addition of HPMC. A PVDF/IL composite film was sandwiched between two AgNW electrodes and sealed using the 3M tape to be used as a transparent pressure sensor.

##### Fabrication of Flexible and Transparent Sensor Array

First, a mask of PI substrate with 3 × 4 or 5 × 5 holes with a diameter of 6.4 mm (3 × 4 array) or 4 mm (5 × 5 array) was made by laser cutting (WE‐6040) and covered on the surface of a bottom PI membrane. AgNW suspension was sprayed on the PI mask, forming a AgNW dot pattern of 3 × 4 or 5 × 5 matrix after removing the mask. After that, thin copper wires were connected to the edges of the AgNW matrix by using conducting resin. This patterned PI/AgNW membrane was used as the bottom electrode for the sensor array. Next, a PVDF/IL composite film was also laser‐cut into pies that aligned with the electrode pattern, and used as the dielectric of the sensors. Another PI/AgNW film acting as the top electrode was then covered on the PVDF/IL dielectric layer, forming a sandwich structure. Finally, the edges of sensor array were sealed by using the 3M tape.

##### Characterizations and Measurements

Micromorphologic images of all specimens were taken by using field‐emission scanning electron microscope (MIRA3, TESCAN). Ultraviolet–visible spectrophotometer (Lambda 950, PerkinElmer) was used to test the optical transmittance of all specimens, and the transparency of the specimens was evaluated based on the transmittance at 550 nm. The transparency of the electrodes and the pressure sensors was evaluated in reference to the PI substrate. The height distribution of porous PVDF and PVDF/IL composite film was analyzed by using a confocal microscope (LSM80C, Carl Zeiss). Mechanical performance of the porous PVDF film and the PVDF/IL composite film was tested by using a tensile machine (XLD‐100E) at loading speed of 5 mm min^−1^ at ambient temperature. The cyclic bending and compression tests were implemented using a home‐made testing system. The capacitance measurements of all sensors were evaluated by using an inductance capacitance and resistance meter (E4980AL, KEYSIGHT) at a testing frequency of 1 × 10^5^ Hz.

##### Optical Simulation

Optical simulations were carried out by using finite element method (FEM). The transmitted spectra were simulated under linear polarized incident light that propagated along the *z*‐axis. The height distribution of PVDF for the simulation was identical with the measured results. The refractive index of PVDF was set to 1.42.

##### Data Transmission System

The data transmission system of the wearable touch sensing consisted of two parts: a data acquisition hardware and a software program in a mobile phone. The data acquisition hardware was made up of a microcontroller unit (MCU), a modulation circuit, and a Bluetooth module, which were combined into a small integral hardware. The MCU generated a signal with a specific frequency to activate the sensor, and data were measured by the modulation circuit. In addition, a channel selector was added to make the system available for the sensor matrix. The Bluetooth module was used to deliver data to the mobile phone through a wireless way. At last, a well‐designed algorithm processed these data and controlled the phone to make a call.

##### Experiments on Human Subjects

The on‐skin experiments were conducted under approval from the Institutional Review Board at the Southern University of Science and Technology (protocol number: 20190007).

## Conflict of Interest

The authors declare no conflict of interest.

## Supporting information

Supporting Information

Supplemental Movie 1

Supplemental Movie 2
