# Supplementary material for: Highly Transparent and Flexible Iontronic Pressure Sensors Based on an Opaque to Transparent Transition
Source: Adv Sci (Weinh). 2020 Mar 12;7(10):2000348. doi: 10.1002/advs.202000348 (PMC7237840; doi:10.1002/advs.202000348)
Supplement: Supplementary file 1 — Supporting Information [file ADVS-7-2000348-s001.pdf]

Copyright WILEY-VCH Verlag GmbH & Co. KGaA, 69469 Weinheim, Germany, 2020.

ADVANCED SCIENCE Open Access

Supporting Information

for *Adv. Sci.,* DOI: 10.1002/advs.202000348

Highly Transparent and Flexible Iontronic Pressure Sensors Based on an Opaque to Transparent Transition

Qingxian Liu, Zhiguang Liu, Chenggao Li, Kewei Xie, Pang Zhu, Biqi Shao, Jianming Zhang, Junlong Yang, Jin Zhang, Quan Wang,* and Chuan Fei Guo**

Supporting Information

Highly Transparent and Flexible Pressure Sensors Based on An Opaque to Transparent Transition

Qingxian Liu, Zhiguang Liu, Chenggao Li, Kewei Xie, Pang Zhu, Biqi Shao, Jianming Zhang, Junlong Yang, Jin Zhang, Quan Wang*, Chuan Fei Guo**

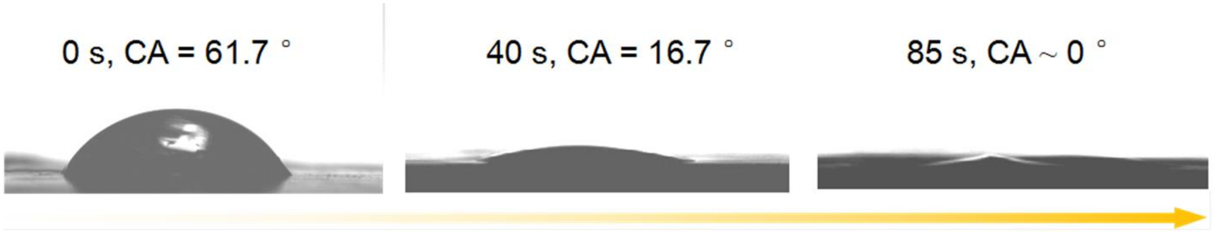

Figure S1. Time-dependent ontact angle (CA) of IL ([BMIM]PF_6) on the surface of the porous PVDF film. It suggests that the [BMIM]PF_6 can immerse inside the porous PVDF film spontaneously.

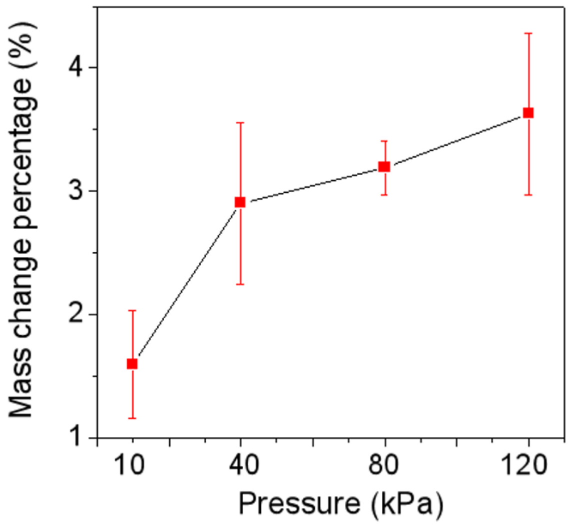

Figure S2. Mass change ratio as a function of pressure for PVDF/IL composite film placed on oleophobic substrate. We can clearly see that the mass loss decreases when put the PVDF/IL composite film on an oleophobic substrate. Under 120 kPa pressure, the mass loss is only 3.6%.

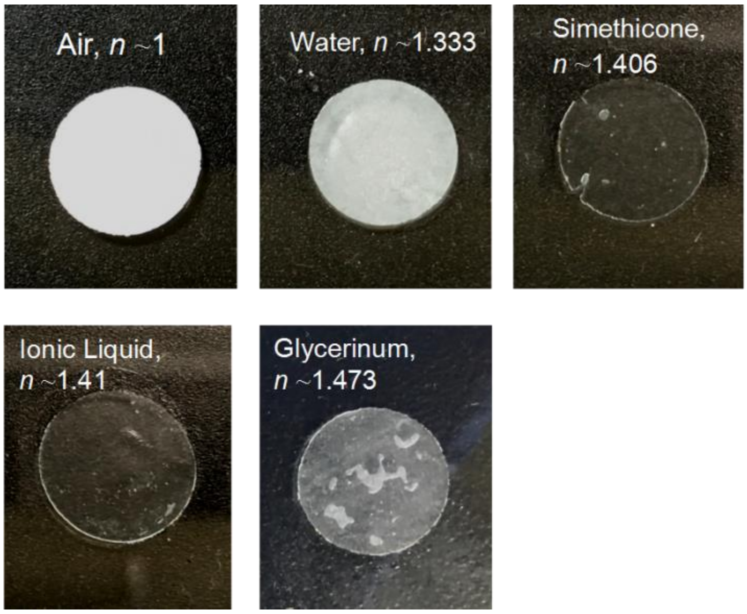

Figure S3. Optical photos of porous PVDF films immersed in various liquids.

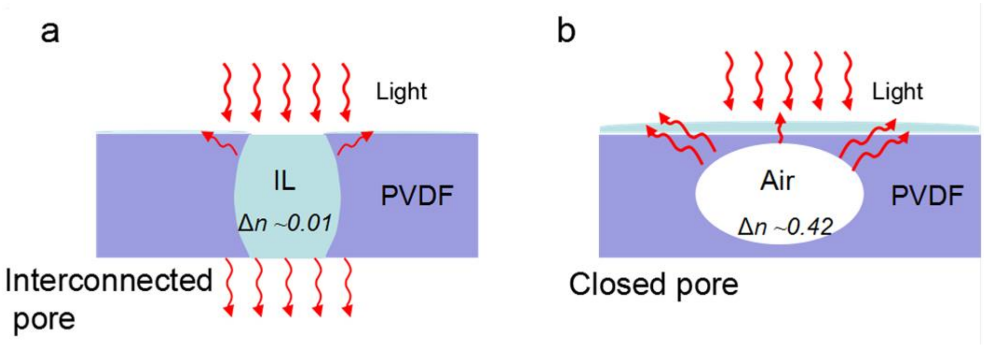

Figure S4. (**a**) Fully interconnected pores are necessary to ensure that the IL can fully infiltrate in the interior of porous PVDF film. Once there are closed pores that IL cannot fill, they will cause strong scattering of light, as illustrated in the schematic (**b**).

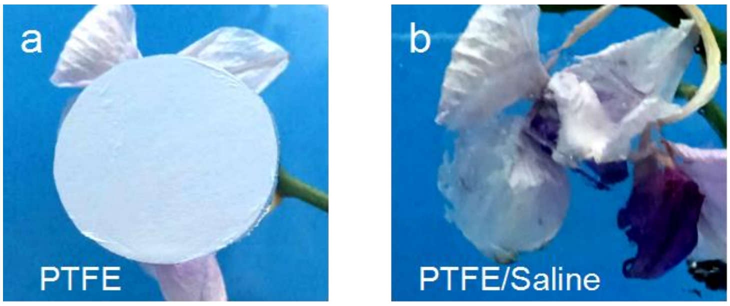

Figure S5. (a) A porous polytetrafluoroethylene (PTFE) film. (b) After immersed in normal saline.

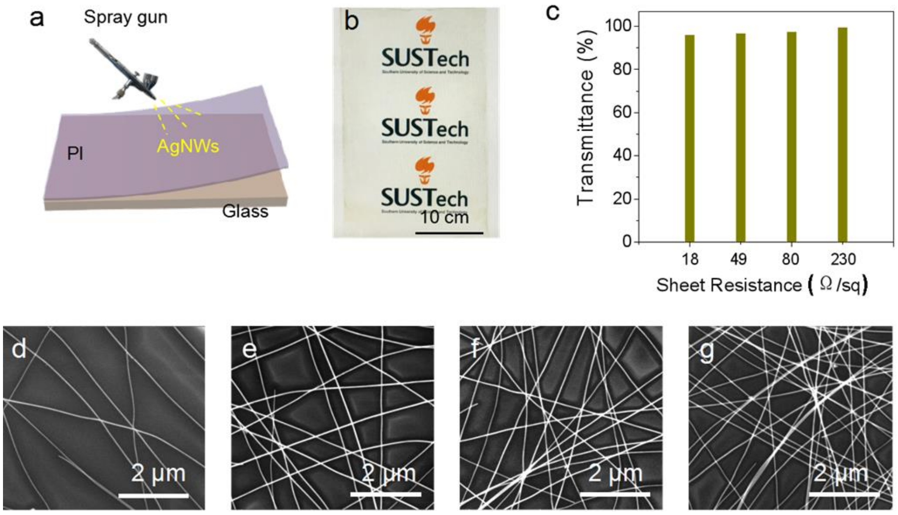

Figure S6. (**a**) Preparation of the transparent electrode by spraying AgNWs on the surface of a PI film. (**b**) Digital photo of the transparent PI/AgNW film with a sheet resistance of 80 Ω/sq. (**c**) The transmittance of AgNW/PI electrodes with various sheet resistances of 18, 49, 80, and 230 Ω/sq. (**d-g**) SEM images of AgNW films with various sheet resistances of (**e**) 18 Ω/sq, (**f**) 49 Ω/sq, (**g**) 80 Ω/sq, and (**h**) 230 Ω/sq.

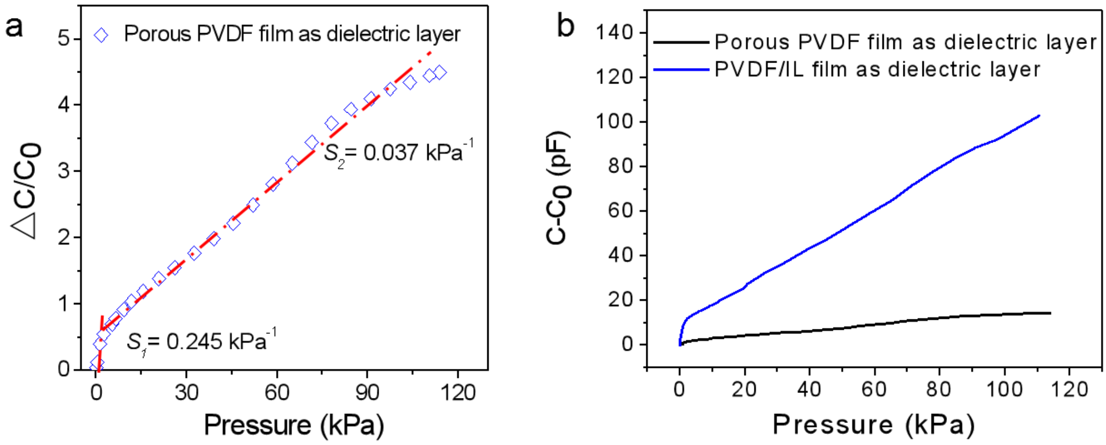

Figure S7. (**a**) Normalized change in capacitance as a function of pressure for the sensor using the original porous PVDF film as the dielectric layer. The sensitivity is lower than that applying the PVDF/IL film as the dielectric layer. (**b**) Comparison of the capacitance variations of the sensors based on the initial porous PVDF dielectric layer and the PVDF/IL dielectric layer.

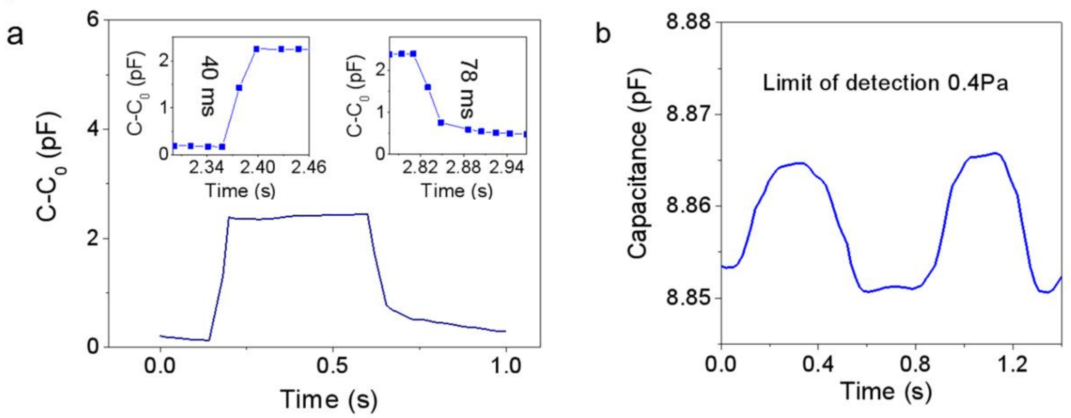

Figure S8. (**a**) Response and relaxion speed of the transparent pressure sensor. The insets show the response and recovery time upon loading and unloading, respectively. (**b**) Limit of detection testing by loading a plastic foam that generates a pressure of 0.4 Pa on the surface of the flexible and transparent sensor.

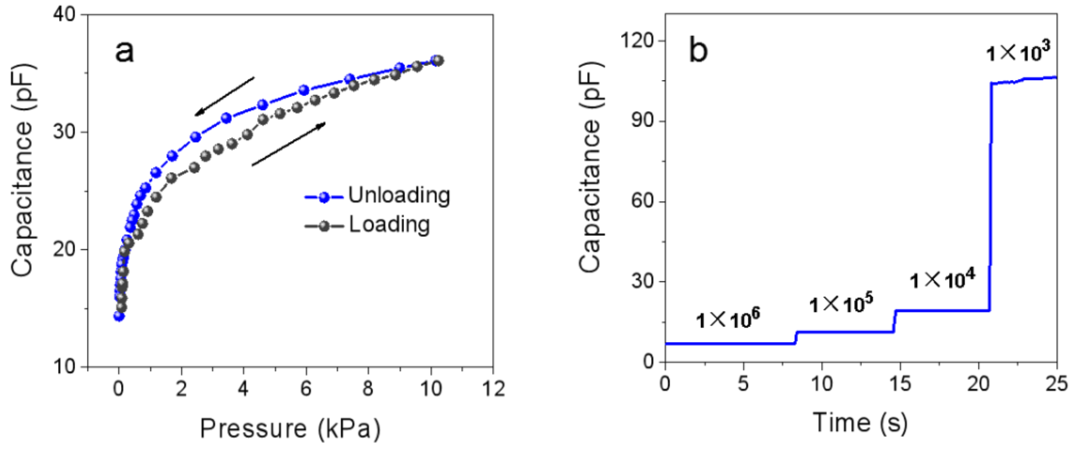

Figure S9. (**a**) Hysteresis of the response under a loading/unloading cycle to 10 kPa. (**b**) Variation of capacitance of the transparent and flexible pressure sensor under various frequencies of 1×10^6 Hz, 1×10^5 Hz, 1×10^4 Hz, and 1×10^3 Hz, indicating an ionic-type behavior.

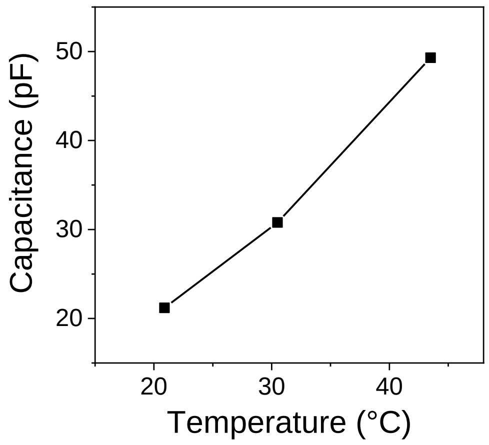

Figure S10. The capacitance signal indensity increases with increasing temperature (from 20.9 to 30.5, and to 43.5 °C).

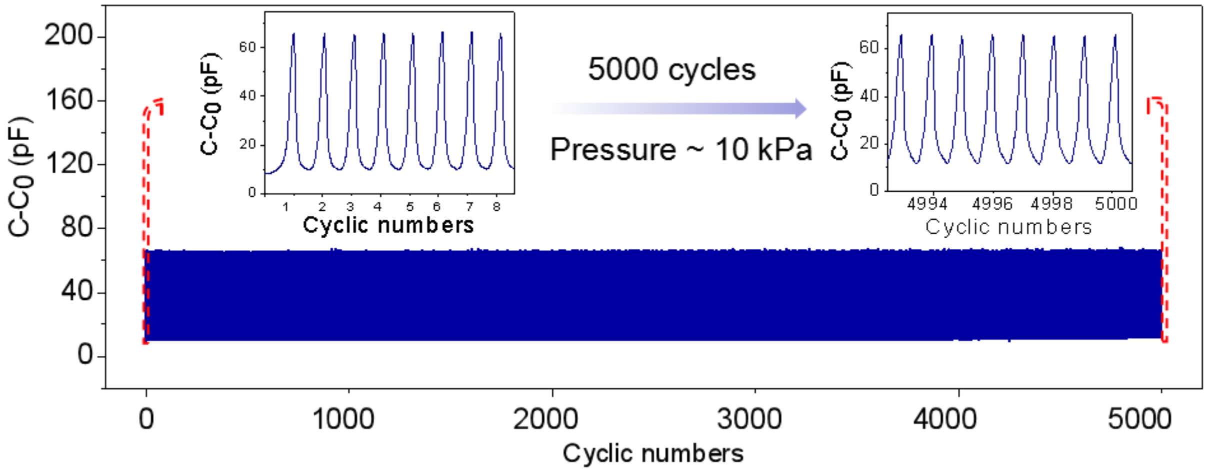

Figure S11. Stability of the flexible and transparent pressure sensor over 5,000 cycles of repeated compression/release at a peak compressive pressure of ~10 kPa. Little change in the signal is observed.

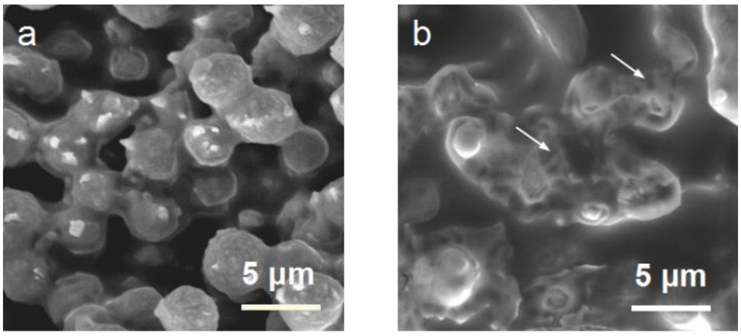

Figure S12. The fracture surface morphology of (**a**) the porous PVDF film and (**b**) the PVDV/IL composite film. The fracture of the original porous PVDF film consists of a great number of microspheres, showing weak residual connections that feature a brittle fracture surface. The fracture surface of the PVDF/IL film has dimples as well as oriented strips along tensile direction, indicating that a significant shear yielding has been occurred, as a result of plasticization caused by the IL. The plasticization effect contributes significantly to energy dissipation and can remarkably improve the toughness of the PVDF film.

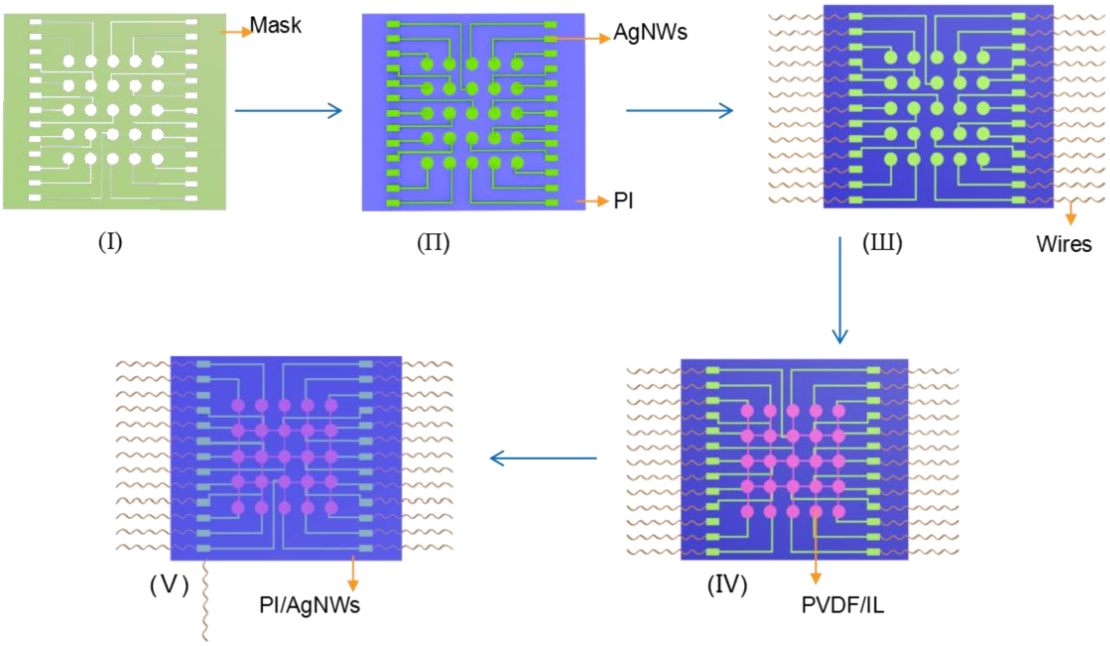

Figure S13. Schematic of the fabrication process of the flexible and transparent sensor array. Details are seen in the Method part.
